# Retraction Note: Blockchain-enabled K-harmonic framework for industrial IoT-based systems

**DOI:** 10.1038/s41598-024-60626-1

**Published:** 2024-05-07

**Authors:** K. M. Baalamurugan, P. Prabu, Nebojsa Bacanin, K. Venkatachalam, S. S. Askar, Mohamed Abouhawwash

**Affiliations:** 1 School of Computing Science and Engineering, Galgotias University, Greater Noida, India; 2 Department of Computer Science, CHRIST (Deemed to be University), Bengaluru, India; 3 Singidunum University, Belgrade, Serbia; 4 Department of Applied Cybernetics, Faculty of Science, University of Hradec Králové, 50003 Hradec Králové, Czech Republic; 5 Department of Statistics and Operations Research, College of Science, King Saud University, P.O. Box 2455, 11451 Riyadh, Saudi Arabia; 6 Department of Mathematics, Faculty of Science, Mansoura University, Mansoura, 35516 Egypt; 7 Department of Computational Mathematics, Science, and Engineering (CMSE), College of Engineering, Michigan State University, East Lansing, MI 48824 USA

Retraction of: *Scientific Reports* 10.1038/s41598-023-27739-5, published online 18 January 2023

The Editors have retracted this Article. After publication, concerns were raised regarding similarities between this Article and previously published work. Specifically, Algorithm 1 in this Article appears highly similar to Algorithm 1 in a previously published paper^1^ with no common authors.

Nebojsa Bacanin agrees with this retraction. K. M. Baalamurugan disagrees with this retraction. Mohamed Abouhawwash did not explicitly state whether or not they agree with this retraction. The remaining Authors did not respond to correspondence from the Editors about this retraction.
